# Supplementary figures and images for: How electronic health literacy influences physical activity behaviour among university students: A moderated mediation model
Source: PLoS One. 2025 Aug 29;20(8):e0330637. doi: 10.1371/journal.pone.0330637 (PMC12396646; doi:10.1371/journal.pone.0330637)

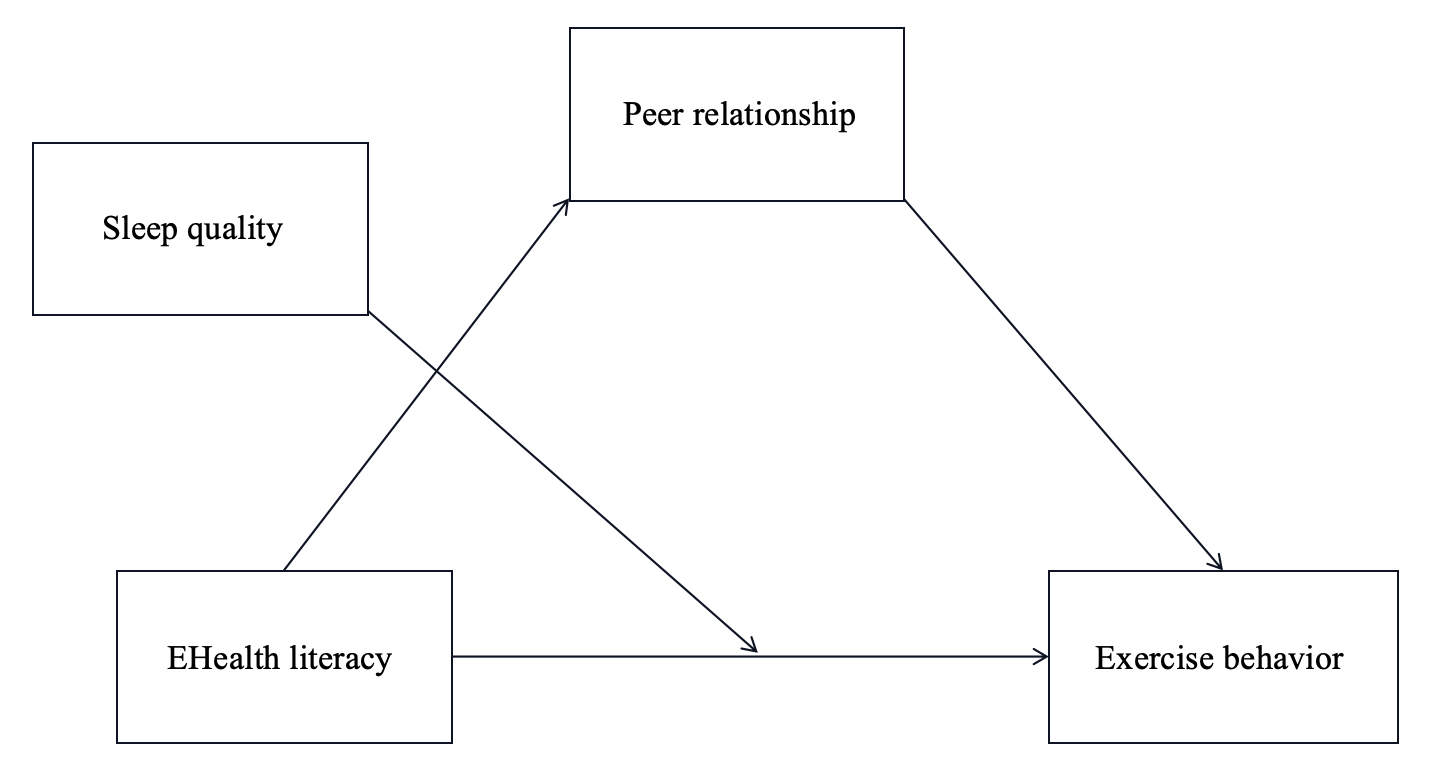

Supplement: S1 Fig — (TIF) [file pone.0330637.s002.tif]

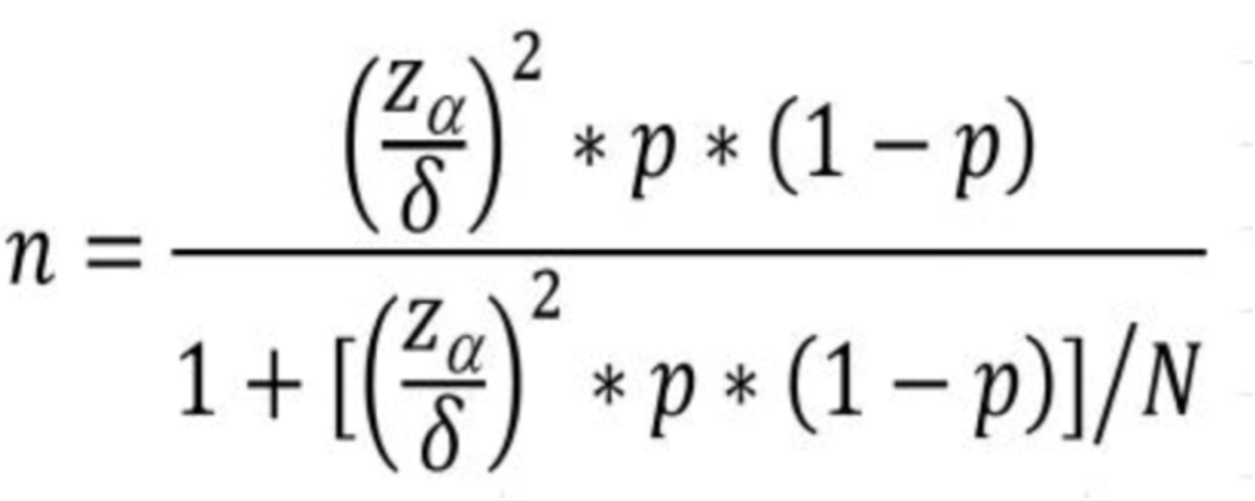

Supplement: S2 Fig — (TIF) [file pone.0330637.s003.tif]

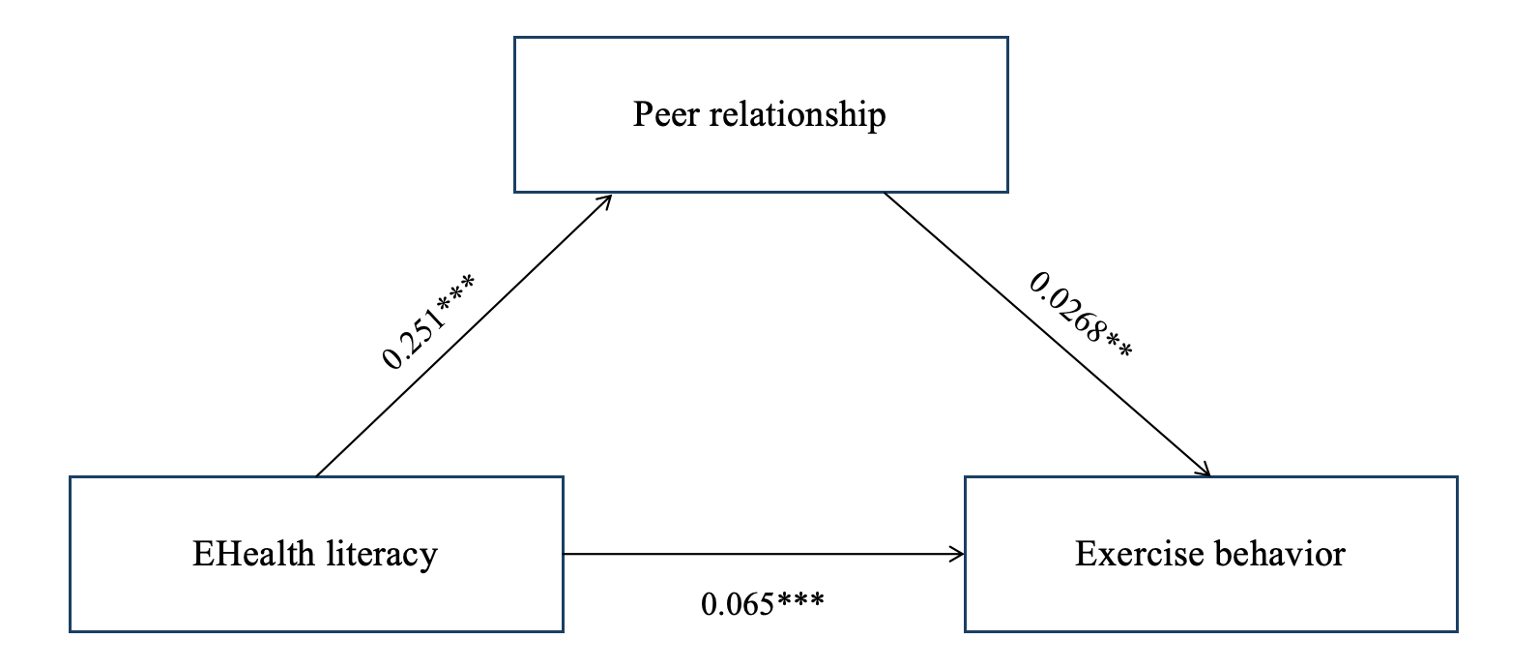

Supplement: S3 Fig — (TIF) [file pone.0330637.s004.tif]

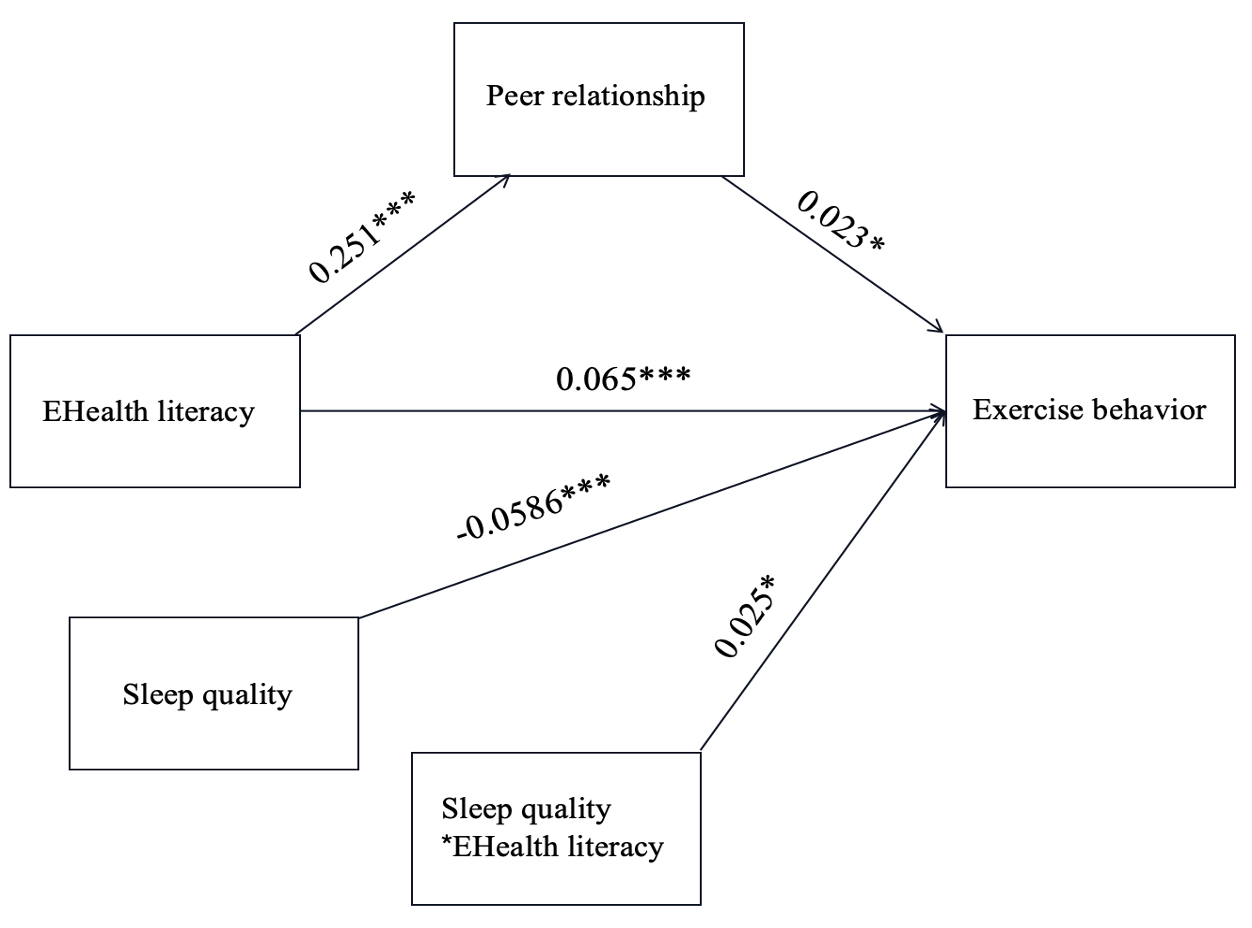

Supplement: S5 Fig — (TIF) [file pone.0330637.s006.tif]
