# Supplementary material for: How electronic health literacy influences physical activity behaviour among university students: A moderated mediation model
Source: PLoS One. 2025 Aug 29;20(8):e0330637. doi: 10.1371/journal.pone.0330637 (PMC12396646; doi:10.1371/journal.pone.0330637)
Supplement: Attachment — Submitted filename: Responses to Editor and Reviewers.docx [file pone.0330637.s008.docx]

Manuscript Number: **PONE-D-25-14894**

Title: **How Electronic Health Literacy Influences Physical Activity Behaviour Among University Students: A Moderated Mediation Mode**

Journal: **PLOS One**

**Point-by-point Responses to Editor**

Dear Editor and dear reviewers,

Thank you very much for your comments and professional advice. These opinions help to improve academic rigor of our manuscript. Based on your suggestion and request, we have made corrected modifications on the revised manuscript. Here are point-by-point responses to your comments, We hope that our work can be improved again.

Sincerely,

On behalf of all authors

**Comment 1:**

Please ensure that your manuscript meets PLOS ONE's style requirements, including those for file naming. The PLOS ONE style templates can be found at 
https://journals.plos.org/plosone/s/file?id=wjVg/PLOSOne_formatting_sample_main_body.pdf and 
https://journals.plos.org/plosone/s/file?id=ba62/PLOSOne_formatting_sample_title_authors_affiliations.pdf

**Response 1:**

**Thank you very much for your detailed review and valuable comments. I have reformatted the manuscript according to PLOS ONE’s style templates, including file naming conventions.**

**Comment 2:**

In the ethics statement in the Methods, you have specified that verbal consent was obtained. Please provide additional details regarding how this consent was documented and witnessed, and state whether this was approved by the IRB.

**Response 2:**

**Thank you for your suggestion! Before the study commenced, all participants were provided with a detailed explanation of the study's purpose, specific procedures, anticipated duration of participation, and any potential risks and discomforts by trained researchers. The researchers ensured that participants fully understood the relevant information and had the opportunity to ask questions to address any concerns. Upon confirming that participants comprehended the information and voluntarily agreed to take part, they expressed their consent orally. Furthermore, this study received approval from the Institutional Review Board (IRB) of Nantong University, with approval number 70 (line 521-528).** **The following documents are specific to informed consent.**

**Comment 3:**

Thank you for stating the following financial disclosure: [2024 General Project of Philosophy and Social Science Research at Universities in Jiangsu Province. (NO：2024SJYB1253)].  
Please state what role the funders took in the study.  If the funders had no role, please state: """"The funders had no role in study design, data collection and analysis, decision to publish, or preparation of the manuscript."""" 
If this statement is not correct you must amend it as needed. 
Please include this amended Role of Funder statement in your cover letter; we will change the online submission form on your behalf.

**Response 3:**

**Thank you very much for your insightful comments and valuable recommendations concerning our manuscript. The funder (2024 Jiangsu Philosophy and Social Science Research Project, No. 2024SJYB1253) only provide financial support for research and distribution of questionnaires, and are not involved in any research activities. The revised funder statement has been included in the cover letter and manuscript.**

**Comment 4:**

We note that your Data Availability Statement is currently as follows: [All relevant data are within the manuscript and its Supporting Information files.]

Please confirm at this time whether or not your submission contains all raw data required to replicate the results of your study. Authors must share the “minimal data set” for their submission. PLOS defines the minimal data set to consist of the data required to replicate all study findings reported in the article, as well as related metadata and methods (https://journals.plos.org/plosone/s/data-availability#loc-minimal-data-set-definition).

For example, authors should submit the following data:

- The values behind the means, standard deviations and other measures reported;
- The values used to build graphs;
- The points extracted from images for analysis.

Authors do not need to submit their entire data set if only a portion of the data was used in the reported study.

If your submission does not contain these data, please either upload them as Supporting Information files or deposit them to a stable, public repository and provide us with the relevant URLs, DOIs, or accession numbers. For a list of recommended repositories, please see https://journals.plos.org/plosone/s/recommended-repositories.

If there are ethical or legal restrictions on sharing a de-identified data set, please explain them in detail (e.g., data contain potentially sensitive information, data are owned by a third-party organization, etc.) and who has imposed them (e.g., an ethics committee). Please also provide contact information for a data access committee, ethics committee, or other institutional body to which data requests may be sent. If data are owned by a third party, please indicate how others may request data access.

**Response 4:**

**Thank you very much for your detailed review and valuable comments. All raw data underlying figures and analyses are provided in Supporting Information (S1_Data). No ethical restrictions apply.**

**Comment 5:**

Please include captions for your Supporting Information files at the end of your manuscript, and update any in-text citations to match accordingly. Please see our Supporting Information guidelines for more information: http://journals.plos.org/plosone/s/supporting-information.

**Response 5:**

**Thank you very much for your insightful comments and valuable recommendations concerning our manuscript.** **I have added captions for figures and tables as required support information section (lines 490-504).**

**Comment 6:**

 Please review your reference list to ensure that it is complete and correct. If you have cited papers that have been retracted, please include the rationale for doing so in the manuscript text, or remove these references and replace them with relevant current references. Any changes to the reference list should be mentioned in the rebuttal letter that accompanies your revised manuscript. If you need to cite a retracted article, indicate the article’s retracted status in the References list and also include a citation and full reference for the retraction notice.

**Response 6:**

**Thank you very much for your insightful comments and valuable recommendations concerning our manuscript. References have been verified. No retracted papers were cited.** **Also, the references cited in this paper are all from the last three to five years with some classic literature**

**Point-by-point Responses to Reviewer 1**

**Comment 1:**

While peer relationships and sleep quality are mentioned as mediators and moderators, respectively, the rationale for their selection and their expected influence on the primary relationship are not detailed in this section.

**Response 1:**

**Thank you very much for pointing out this issue, which is extremely important for enhancing the quality and applicability of our research.** **Sleep was chosen as a moderating variable because it forms the physiological basis for health information processing, while peer relationships bridge the knowledge-behaviour transformation through social support mechanisms. We added justifications in the Introduction (Lines 101-105) for peer relationships and sleep quality influence on the primary relationship.**

**Comment 2:**

The selection of universities and the rationale behind choosing these specific institutions are not explained, which
could impact the representativeness of the sample.
- There is a lack of detail on the data collection process.
- The methodology used to handle missing data is not mentioned.
- Established criteria were not mentioned other than the age (line 163).
- Sampling technique is not clear

**Response 2:**

**Thank you very much for your detailed review and valuable comments.**

**First, selection criteria for the institutions: 1) Regional representation (East China / Central China); 2) Coverage of types of education (comprehensive universities / normal universities / application-oriented undergraduate institutions / vocational colleges); 3) Exclusion of sports-related institutions to avoid sample bias. (line 188-191).**

**Second,** **the detailed data collection process has been added in lines 155-184.**

**Third,** **this study established certain standards during data collection, thus there are no missing data that need further processing.**

**Forth,** **① Delete questionnaires completed by respondents aged under 18 or over 23; ② Exclude incomplete questionnaires or those with missing responses; ③ Remove entries with contradictory or conflicting options; ④ Discard multiple submissions from the same IP/ID; ⑤ excluding questionnaires that did not follow the instructions and had consistent answers to all questions, resulting in a total of 913 questionnaires being removed.** **The above data screening criteria have been added in lines 198-206.**

**Fifth,** **we used stratified purposive sampling with a sample covering the four main types of Chinese HEIs (37.5% comprehensive/teacher-training 30.9%/applied 24.3%/higher vocational 7.3%), which was not significantly different from the national distribution of types of HEIs (Ministry of Education 2023: 40.1%/28.7%/22.5%/8.7%) (χ²=3.21, p=0.36)**

**Comment 3:**

The conclusion lacks a critical reflection on the limitations of the study.

**Response 3:**

**Thank you very much for pointing out this issue, which is extremely important for enhancing the quality and applicability of our research.** **This study has three limitations: 1) The cross-sectional design makes it difficult to infer causal relationships over time; future research should employ cross-lagged models for tracking; 2) The sample does not cover higher education institutions in the western regions, which limits the generalisability of the conclusions; and 3) Potential confounding variables, such as family economic status, were not controlled for.The limitations of this study have been included in the conclusion section (lines 489-494).**

**Point-by-point Responses to Reviewer 2**

**Comment 1:**

The abstract provides a clear and concise summary of the research and effectively highlights the moderated mediation model. However, it would benefit from briefly indicating the study design and sampling technique to provide readers with essential methodological context.

**Response 1:**

**We have clarified the stratified purposive sampling methodology in the abstract (line 46-52) to address methodological concerns.**

**Comment 2:**

The introduction successfully conveys the relevance of eHealth literacy and its potential influence on exercise behavior among university students. The literature review is well-structured and informative. Clarification is needed regarding the choice of sleep and peer relationships as the mediating and moderating variables (lines 96–97), as well as the rationale behind the statement about the lack of a systematic analysis of linking mechanisms (line 95).

**Response 2:**

**Thank you for your valuable comments on the statistical analysis section of our paper. Sleep was chosen as a moderating variable because it forms the physiological basis for health information processing, while peer relationships bridge the knowledge-behaviour transformation through social support mechanisms. Together, these factors help to explain the boundary conditions and pathways through which electronic health literacy affects behaviour.** **We have provided the reasons for selecting peer relationships and sleep quality as mediating and moderating variables in line line 101-105.**

**Comment 3:**

The methodology section is detailed and offers a comprehensive account of data collection, measurement instruments, and statistical techniques. The use of established scales (e.g., eHealth Literacy Scale, Physical Activity Rating Scale) strengthens the study’s reliability. However, the explanation of the study design and sampling strategy lacks clarity. It is recommended to elaborate on the rationale behind the university selection criteria specifically, whether institutions focusing on health and physical education were included. Additionally, the specific sampling technique used should be clearly stated. These clarifications would support a better understanding of the study’s generalizability.

**Response 3:**

**Thank you for your meticulous review and valuable feedback on our paper. Selection criteria for the institutions: 1) Regional representation (East China / Central China); 2) Coverage of types of education (comprehensive universities / normal universities / application-oriented undergraduate institutions / vocational colleges); 3) Exclusion of sports-related institutions to avoid sample bias.**

**Comment 4:**

The discussion provides a thoughtful interpretation of the results, placing them effectively within the context of existing literature. The articulation of the study’s contributions is clear, and the acknowledgment of limitations is comprehensive. To strengthen this section, more specific suggestions for future research could be added. Additionally, while theoretical implications are well-addressed, practical applications particularly how findings can inform interventions to promote physical activity in university populations deserve more attention.

**Response 4:**

**Thank you very much for pointing out this issue, which is extremely important for enhancing the quality and applicability of our research. Research indicates that physical exercise demonstrates interconnected health behaviours by improving university students' sleep quality. Exercise not only alleviates social anxiety and mobile phone dependency but also indirectly enhances sleep through physiological and psychological regulation. Concurrently, higher e-health literacy enables students to acquire scientific exercise knowledge, bolsters their motivation for physical activity, and strengthens behavioural execution – thereby reinforcing exercise engagement at a cognitive level. These factors create a synergistic effect in promoting sleep. (line 469-476).**

**Comment 5:**

The conclusion effectively summarizes the study’s findings and their alignment with the original research objectives. The emphasis on the role of eHealth literacy in shaping exercise behavior, mediated by peer relationships and moderated by sleep quality, is well-articulated. To further enhance the impact of this section, consider expanding on potential practical recommendations derived from the findings.

**Response 5:**

**Thank you very much for your valuable comments and suggestions. We have considered your request regarding the revision of the conclusions. However, we would like to state for the record that conclusions are based on results Altering the conclusions might affect the coherence and integrity of the research narrative. We believe that the current conclusions accurately reflect the findings and implications of the study was conducted. We truly appreciate your understanding of this matter.**
